# Applications of machine and deep learning to thyroid cytology and histopathology: a review

**DOI:** 10.3389/fonc.2023.958310

**Published:** 2023-11-07

**Authors:** Greg Slabaugh, Luis Beltran, Hasan Rizvi, Panos Deloukas, Eirini Marouli

**Affiliations:** ^1^ Digital Environment Research Institute, Queen Mary University of London, London, United Kingdom; ^2^ Barts Health NHS Trust, The Royal London Hospital, London, United Kingdom; ^3^ William Harvey Research Institute, Barts and The London School of Medicine and Dentistry, Queen Mary University of London, London, United Kingdom

**Keywords:** thyroid cancer, cytopathology, histopathology, machine learning, deep learning, artificial intelligence

## Abstract

This review synthesises past research into how machine and deep learning can improve the cyto- and histopathology processing pipelines for thyroid cancer diagnosis. The current gold-standard preoperative technique of fine-needle aspiration cytology has high interobserver variability, often returns indeterminate samples and cannot reliably identify some pathologies; histopathology analysis addresses these issues to an extent, but it requires surgical resection of the suspicious lesions so cannot influence preoperative decisions. Motivated by these issues, as well as by the chronic shortage of trained pathologists, much research has been conducted into how artificial intelligence could improve current pipelines and reduce the pressure on clinicians. Many past studies have indicated the significant potential of automated image analysis in classifying thyroid lesions, particularly for those of papillary thyroid carcinoma, but these have generally been retrospective, so questions remain about both the practical efficacy of these automated tools and the realities of integrating them into clinical workflows. Furthermore, the nature of thyroid lesion classification is significantly more nuanced in practice than many current studies have addressed, and this, along with the heterogeneous nature of processing pipelines in different laboratories, means that no solution has proven itself robust enough for clinical adoption. There are, therefore, multiple avenues for future research: examine the practical implementation of these algorithms as pathologist decision-support systems; improve interpretability, which is necessary for developing trust with clinicians and regulators; and investigate multiclassification on diverse multicentre datasets, aiming for methods that demonstrate high performance in a process- and equipment-agnostic manner.

## 1 Introduction

### 1.1 Thyroid cancer

The thyroid is a small, butterfly-shaped gland in the neck on which nodules – small fluid or solid lumps – can develop. These nodules are detectable with palpation in about 2–6% of the population and with ultrasound in around 19–67% (1). Most are benign, but approximately 5% are cancerous (2).

There are four main types of thyroid cancer – papillary (PTC), follicular (FTC), anaplastic (ATC) and medullary (MTC) thyroid carcinoma (3) – which account for 70–90%, 5-10%, 2% and <2% of cases, respectively (4–6)Variants/subtypes exist, such as the follicular or tall cell variants of PTC. An additional diagnosis of particular note is that of ‘noninvasive follicular thyroid neoplasm with papillary-like nuclear features’ (NIFTPs). This term was introduced in 2016 to replace the noninvasive encapsulated follicular variant of PTC (EFV-PTC): despite NIFTPs having cells resembling those of PTC, they have low malignant potential, and effectively distinguishing between NIFTPs and classic PTC can aid in therapy de-escalation (3, 7, 8).

Benign nodules are usually represented by non-neoplastic (multinodular hyperplasia/goitre, cysts) and neoplastic [follicular adenoma (FA)] lesions.

#### 1.1.1 Diagnosis and treatment

Generally, thyroid nodules are discovered incidentally during a routine health check, through clinical examination of another condition, or by the patient (9). Nodules are typically investigated using ultrasound first to check composition, size, location, echogenicity and calcification amongst other parameters (3, 10), and depending on the results, patients may be recommended for fine-needle aspiration (FNA) cytology. The latter is the gold-standard preoperative diagnosis technique and has a reported sensitivity and specificity of 68–98 and 56–100%, respectively (11).

The Bethesda System (TBS) for Reporting Thyroid Cytopathology is a widely adopted reporting system for FNA samples (**Table 1**) (10, 12). National adaptations exist to cater to local need, such as the Thy1–5 system used in the UK (14), with broad equivalences between systems. Around 10% of acquired FNAs are unsatisfactory for diagnosis (TBS1) due to parameters like obscuring blood, poor cell preservation, and insufficient cell sampling (15, 16), with the recommendation to repeat the biopsy with ultrasound guidance. For some malignancies cytological diagnosis is challenging (17–20), resulting in both high interobserver variability (21, 22) and an estimated 15–30% returning an indeterminate result (TBS3 or 4) (11, 16). For such a finding, the respective risk of malignancy were given in a meta-analysis as up to 30.5% for TBS3 and up to 28.9% for TBS4 (13) (15). The relatively high risk often motivates diagnostic surgery in the form of either a lobectomy or thyroidectomy (10, 23), with analysis of the excised tissues the gold standard for diagnosis. Surgical excision is the recommended course of action for malignant nodules, but a large proportion of diagnostic surgeries are evidently unnecessary. Surgery carries risks (24), can cause substantial distress and anxiety, and in many cases necessitates lifelong thyroxine replacement therapy (10). Many patients experience this needlessly.

**Table 1:** The Bethesda System for reporting thyroid cytopathology (12).

Category	Diagnostic Category	Predicted TBS Risk of Malignancy if NIFTP ≠ CA (%)	Risk of malignancy when non-invasive follicular thyroid neoplasm is considered cancer.	Usual Management^a^
**TBS1**	Nondiagnostic or unsatisfactory	5–10	2.0-19.1	Repeat FNA with ultrasound guidance
**TBS2**	Benign	0–3	0.7-8.0	Clinical and sonographic follow up
**TBS3**	Atypia of undetermined significance or follicular lesion of undetermined significance	6–18	9.2-30.5	Repeat FNA, molecular testing, or lobectomy
**TBS4**	Follicular neoplasm or suspicious for a follicular neoplasm	10–40	28.9	Molecular testing, lobectomy
**TBS5**	Suspicious for malignancy	45–60	79.6	Near-total thyroidectomy or lobectomy
**TBS6**	Malignant	94–96	99.1	Near-total thyroidectomy or lobectomy

a Actual management may depend on other factors (e.g. clinical, sonographic) besides the FNA interpretation. NIFTP, noninvasive follicular thyroid neoplasm with papillary-like nuclear features; CA, carcinoma; FNA, fine-needle aspiration. Risk of malignancy are from a meta-analysis conducted by Huy Gia Vuong et al. (13).

Furthermore, manual analysis of the biopsy and tissue samples is laborious, with the time pressure it places on pathologists exacerbated by increased workloads and the chronic shortage of trained staff (25). Better methods of malignancy prediction are necessary throughout the diagnostic pipeline to alleviate this pressure, decrease the number of unnecessary surgeries, and improve general patient well-being.

Molecular testing has been proposed to augment malignancy prediction for cytologically indeterminate nodules, with many studies reporting success (26–29). While such methods certainly have a place in thyroid cancer diagnosis, concerns exist that these tests generally reduce the risk of cancer presence rather than guarantee its absence (30), are restricted to few highly specialised and centralised laboratories (31), and augment the total cost of healthcare (32), which inhibits clinical adoption in resource-constrained settings.

Motivated by the above, much research has been conducted into how techniques utilising artificial intelligence (AI) may improve the existing clinical workflow.

### 1.2 Artificial intelligence

#### 1.2.1 Background

AI is a field that involves teaching computers and machines how to make decisions and solve problems intelligently (33). Historically, it was concerned with computationally reproducing capabilities of the human brain, although modern AI is less focused on mimicking biological processes and more about solving complex problems regardless of biological inspiration (34).

Machine learning (ML) is a branch of AI defined as the study of computer algorithms that automatically improve through experience (35). Data is used to train these algorithms to perform a task – for example, regression, classification or clustering – in a way that optimises some performance metric without the need for explicit programming.

Deep learning (DL) is a branch of ML adept at automatically discovering patterns directly from raw data (36). It concerns the application of neural networks – the learning is ‘deep’ as these networks comprise many layers that in turn consist of many computational neurons – and has demonstrated high performance at tasks involving unstructured data, such as image analysis (37) and speech recognition (38).

AI research has increased in recent years due to the greater availability of large datasets, improved processing power – particularly with the introduction of graphics processing units (GPUs) for massively parallel computation (39) – and the increased availability of open-source software libraries that ease algorithm implementation.

#### 1.2.2 Biomedical applications

ML and DL are particularly applicable to biomedicine, as they can be used to discover patterns unseen by humans (such as in drug discovery and genetic analysis), assist biomedical image analysis to reduce the pressure on clinicians, and predict outcomes from clinical data (40). For instance, they have been employed to estimate unknown bio-interactions between drug compounds and biological targets (41), predict adverse events in drug discovery (42), predict sequence specificities in DNA- and RNA-binding proteins (43, 44), automate the interpretation of echocardiograms (45), automate the classification of organ- or body part-specific images (46), and both screen for (47, 48), and predict mortality and hospitalisation in, heart failure (49).

ML and DL have also been applied successfully to many other cancers: to automatically classify nodules from CT images (50) and predict the presence of mutations from histopathology images (51) in lung cancer, to predict axillary lymph node status from ultrasound images (52) and link tumour morphology and spatially localised gene expression from histopathology images (53) in breast cancer, and to classify and segment suspicious lesions from MRI (54) and automate Gleason grading of biopsies (55) in prostate cancer.

Within thyroid cancer, ML and DL have been applied to other imaging modalities: to diagnose cervical lymph node metastasis in CT images (56) and for computer-aided diagnosis and risk stratification of thyroid nodules in ultrasound scans (57–59). Although thyroid cancer evaluation is mainly concerned with image analysis, models also exist for diagnosis (26–29), risk stratification (57) and prediction of lymph node metastases (58) from DNA- and RNA-sequencing data.

This review shall focus on the application of ML and DL to thyroid cancer cyto- and histopathology. Research studies were identified by searching PubMed with terms including thyroid cancer, machine learning, deep learning and artificial intelligence. Further studies were identified from references within suitable papers and reviewer recommendation. In order to capture the recent literature only studies published since 2017 have been summarised, with references to older papers provided.

## 2 Current applications

**Figure 1:**
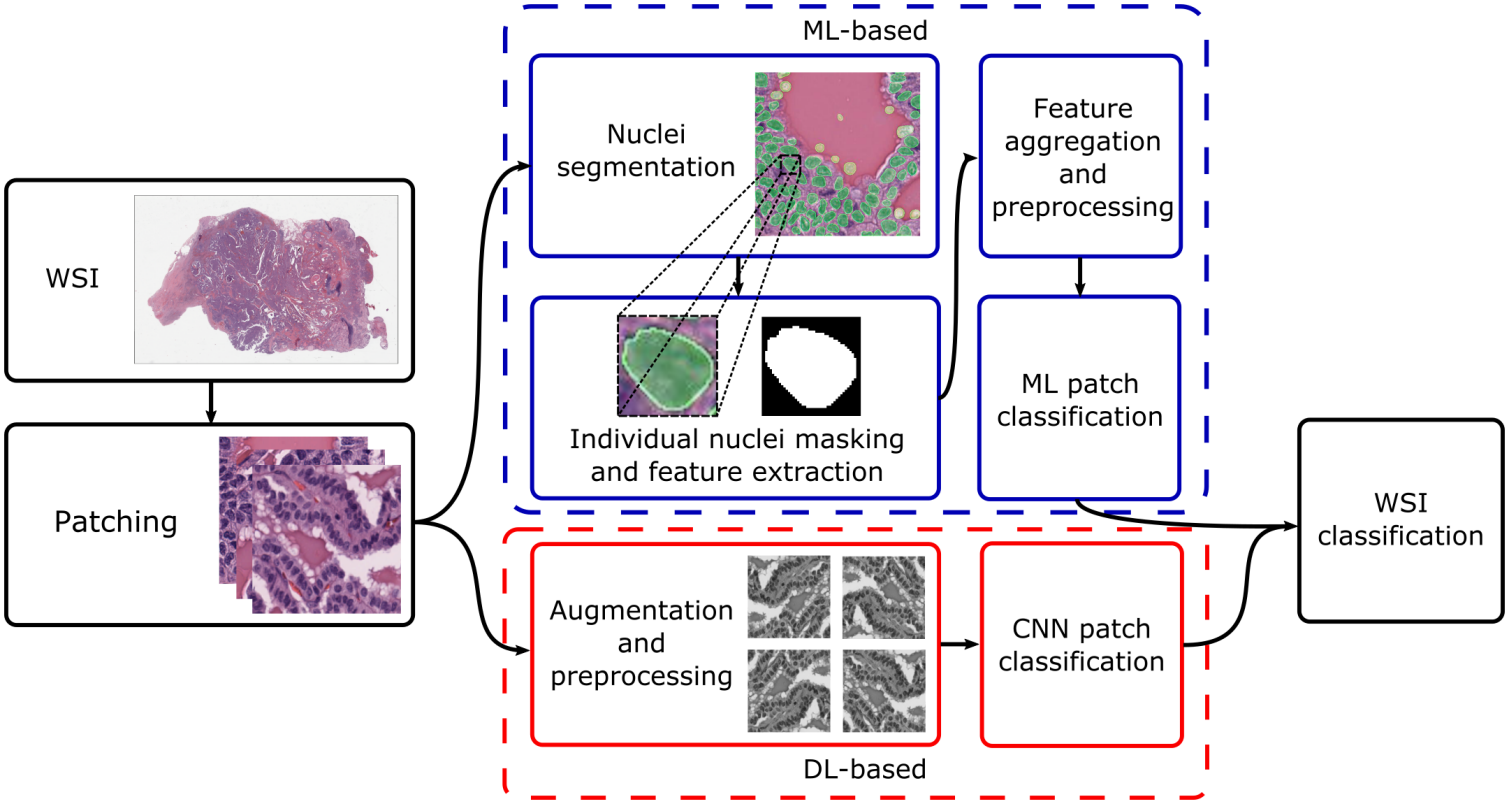
Typical processing pipelines. The WSI was taken from The Cancer Genome Atlas (project ID: TCGA-THCA), and nuclei segmentation was performed using nucleAIzer (59). WSI, whole-slide image; ML, machine learning; DL, deep learning; CNN, convolutional neural network.

Current research concentrates on thyroid nodule classification. This can be broad – for instance, classifying nodules as benign or malignant – or more granular, with differential diagnoses given for the specific nodule type. Broadly, approaches for both cyto- and histopathology can be split based on whether they utilise traditional ML or DL; **Figure 1** shows typical processing pipelines for digitised whole-slide images (WSIs) in both cases. The WSI is first patched into smaller cell regions; this can be done by a pathologist, who may highlight informative regions and annotate them (as, for instance, benign or suspicious for malignancy), or automatically, where usually the slide-level diagnosis is cascaded down to the patches. For ML-based pipelines, the nuclei are then segmented (which may utilise DL or other techniques), their features are extracted and aggregated, and an ML algorithm is used to classify the patch based on the feature values. For DL-based pipelines, datasets are generally augmented before being fed into a convolutional neural network (CNN). Local patch-level classifications can then be aggregated into a global WSI-level diagnosis.

Other research areas include the effective segmentation of follicular cells, evaluation of how screening software may improve pathologist workflow, prediction of both genetic mutational status and lymph node metastases.

### 2.1 Cytopathology

**Table 2** summarises research published since 2017 that applies AI to thyroid cancer cytopathology. The aims vary between studies – for example, some try to distinguish between benign and malignant nodules of all types, while others focus on differentiating FTC from FA – as do the techniques employed, the natures and sizes of the datasets, and whether evaluation metrics are calculated on a slide/patient or extracted image level. The results are therefore not directly comparable but have nevertheless been provided.

**Table 2:** A summary of recent research applying AI to thyroid cytology specimens.

Study	Year	Aim	Technique	Level	Sample Size	Reported Metrics	Results
Savala et al. (60)	2018	FTC vs FA	Neural network	Slide	57	Accuracy	100%
Margari et al. (61)	2018	Predict TBS diagnosis	Classification and regression trees	Slide	521	Accuracy	91%
		Benign vs malignant				Accuracy Sensitivity Specificity	93.0% 92.4% 93.6%
Sanyal et al. (62)	2018	PTC vs non-PTC	CNN	Image	370	Accuracy Sensitivity Specificity	85.1% 90.5% 83.3%
Guan et al. (63)	2019	PTC vs benign	CNN	Slide	279	Accuracy	95.0%
				Image	887	Accuracy Sensitivity Specificity	97.7% 100% 94.9%
Maleki et al. (64)	2019	PTC vs NIFTPs and noninvasive EFV-PTC	Support vector machine	Slide	59	Accuracy Sensitivity Specificity	76.1% 72.6% 81.6%
Fragopoulos et al. (65)	2020	Benign vs malignant	Neural network	Slide	447	Accuracy Sensitivity Specificity	95.1% 95.0% 95.1%
Elliott Range et al. (66)	2020	Benign vs malignant	Two CNNs	Slide	908	Sensitivity Specificity AUROC	92.0% 90.5% 0.932
Zhu et al. (67)	2021	Efficient follicular cell segmentation	CNN	Slide	43	Pixel Accuracy	99.3% in 49.5 s
				Image	6,900	Pixel Accuracy	98.7% in 97.4 s
Lin et al. (68)	2021	Fast segmentation of PTC	CNN	Slide	131	Accuracy Precision Recall	99% 86% 94%
Dov et al. (69)	2021	Benign vs malignant	Two CNNs	Slide	908	AUROC Average Precision	0.870 74.3%
Yao et al. (70)	2022	Benign vs FA	Gradient boosting and extra trees classifiers	Image	800	AUROC Accuracy Precision Recall	0.75 71% 72% 71%
Dov et al. (71)	2022	Assess pathologist performance when using and not using a decision-support system	Screening software utilising two CNNs	Slide	109	Pairwise weighted kappa statistic	0.924

FTC, follicular thyroid carcinoma; FA, follicular adenoma; TBS, The Bethesda System; PTC, papillary thyroid carcinoma; CNN, convolutional neural network; NIFTP, noninvasive follicular thyroid neoplasm with papillary-like nuclear features; EFV-PTC, encapsulated follicular variant of papillary thyroid cancer; AUROC, area under the receiver operating characteristic curve.

The level column describes whether metrics were calculated for full slides or extracted images.

Older papers from 1996–2014, where less advanced and sophisticated methods were employed include Karakitsos et al. (72, 73), Harms et al. (74), Ippolito et al. (75), Cochand-Priollet et al. (76), Shapiro et al. (77), Daskalakis et al. (78), Zoulias et al. (79), Varlatzidou et al. (80), Gopinath et al. (81–84), Saylam et al. (85) and Huang et al. (86).

#### 2.1.1 Machine learning-based methods

##### 2.1.1.1 Classification of carcinomas

Margari et al. (61) used classification and regression trees (CARTs) to evaluate thyroid lesions and extract human-understandable knowledge of the diagnostic process. The study included 521 cases of benign (261), malignant (256) and indeterminate (4) nodules confirmed using histology. Categorical cytomorphological characteristics were extracted and used to develop two models: CART-C for predicting TBS category and CART-H for the histological diagnosis of benign or malignant. CART-C achieved 91.0% accuracy when predicting TBS category; if TBS3 was used as a cut-off to classify nodules as either benign or malignant, CART-C achieved a respective sensitivity and specificity of 88.5% and 79.7%, and if TBS4 was used, the same values were 78.1% and 94.4%. These results were not statistically different from those of pathologists. CART-H achieved 93.0% accuracy, 92.4% sensitivity and 93.6% specificity when predicting the histological diagnosis.

Maleki et al. (64) worked to differentiate classic PTC from NIFTPs and noninvasive EFV-PTC using a support vector machine (SVM) trained on textual descriptions. Surgical pathology cases with one of the diagnoses, an FNA matching the tumour, and an available microscopic description were selected, which resulted in 59 cases (29 PTC, 30 NIFTP/EFV-PTC). A total of 59 different cytomorphological phrases were extracted from the microscopic descriptions; these were condensed into 32 categories (with, for instance, oncocytic cells and oncocyte reduced to one category). The classifier was then trained using all cases except for one randomly selected case each of PTC and NIFTP, which were used for evaluation; this was done for all possible iterations of excluded pairs. The classifier achieved 76.1% accuracy, 72.6% sensitivity and 81.6% specificity. Key phrases associated with NIFTPs were ‘scant colloid’, ‘microfollicular pattern’, ‘oncocyte’, ‘crowded’ and ‘small follicular cell’; those associated with PTC were ‘papillary’, ‘pale chromatin’, ‘focal’, ‘prominent nucleoli’, and ‘nuclear groove’.

##### 2.1.1.2 Other Research Areas

Yao et al. (70) evaluated ThinPrep, an existing preparation technique optimised for digital pathology and ML algorithms, as a means of improving diagnostic accuracy and reproducibility for TBS3 cases. Their study used 40 FNAs (20 benign, 20 FA) confirmed with histology; morphological differences between these two cases are known to be more subjective, with less reproducible diagnoses. A total of 800 images were extracted – 20 from each case, 10 mid-power (100x) and 10 high-power (400x) – with each image reviewed by a cytopathologist to evaluate adequate cellularity and assign a TBS category. Traditional computer vision-based techniques were used to extract 86 low-level nuclear features, and these were grouped to form 3 mid- and 3 high-level features based on the authors’ cytomorphological knowledge. Gradient boosting and extra trees classifiers were trained separately on the mid- and high-power magnifications. The top-performing model was trained on the mid-power set and achieved 71% accuracy, 72% precision and 71% recall on the test set, with FA considered the positive class. By comparison, the cytopathologist achieved 63% accuracy, 57% precision and 95% recall, indicating a more cautious approach at the expense of broader accuracy.

Since the advent of CNNs and the subsequent growth of DL-based approaches for cytological classification, authors utilising traditional ML have applied it in less conventional cases, such as through utilising CARTs and text-based features instead. The moderate success of Maleki et al. (64) and Yao et al. (70) suggest that ML has some potential to address difficult preoperative challenges, although improvements remain necessary.

#### 2.1.2 Deep learning-based methods

##### 2.1.2.1 Classification of carcinomas

Savala et al. (60) employed a neural network to differentiate cases of FA (26) and FTC (31). Images prepared with two different WSI stains – May Grünwald-Giemsa (MGG) and haematoxylin and eosin (H&E) – were included, and histology was used as the gold standard, where 90% of FTC cases were found to be minimally invasive. Single-cell images were extracted manually – around 100 for each case – individual nuclear features were then computed with image processing software, and mean values for various morphometric and densitometric features were included for each collective sample. The validation and test sets contained nine samples each; the former was classified with an accuracy of 78%, sensitivity of 75% and specificity of 80%, and the latter was classified perfectly. Despite this perfect test classification, further investigation is required: the test set was small, and the large discrepancy between the results achieved on the same-sized validation and test sets highlights the natural variance expected at such scales.

Sanyal et al. (62) used a CNN to classify PTC and non-PTC samples. Only cases where a cytological diagnosis was reached were included, and diagnoses were confirmed with histology; borderline cases were excluded. For model training, 20 cytology slides (either Romanowsky- or Papanicolaou-stained) were selected from two different centres, and two different digital microscopes were used to extract 370 512x512 pixel images (184 PTC, 186 non-PTC; 209 at 10x and 161 at 40x magnification) focusing on diagnostic areas of interest. A separate test set was developed by selecting 87 regions from ten smears that displayed either PTC (21) or non-PTC (78) behaviour, with all regions photographed at 10x and 40x magnification. The CNN classified these images separately, and when using or-based decision criteria (where, if a sample was classified as PTC at either magnification, the sample-level classification was PTC), the CNN achieved an image-level accuracy of 85.1%, sensitivity of 90.5% and specificity of 83.3%.

Guan et al. (63) employed a VGG16-based CNN via transfer learning to differentiate PTC from benign thyroid nodules. The study included 279 H&E-stained cytological slides (159 PTC, 120 benign). All PTC cases were classified as either TBS5 or 6 and had typical PTC features and a histologically confirmed diagnosis; all benign images were classified as TBS2, but the patients did not undergo surgery, so histological diagnosis was unavailable. Each larger image was manually segmented into several smaller 224x224 pixel fragments that contained the cells, which gave 887 images in total (476 PTC, 411 benign), and this dataset was augmented by a factor of eight through flips and rotations. The CNN achieved 97.7% accuracy, 100% sensitivity and 94.9% specificity on an image level and 95% accuracy on a patient level. Nuclear features were also automatically extracted and compared with t-tests; the number of contours, the cell perimeter and area, and mean pixel intensity were all statistically bigger for malignant cells. A limitation of the study is that it only included slides categorised as TBS2, 5 or 6 – images that FNA would generally catch – and the authors advised that future studies investigate CNN performance on indeterminate cases (TBS3 or 4) and other types of thyroid cancer.

Fragopoulos et al. (65) implemented a neural network to classify liquid-based cytology WSIs as either benign or malignant. The study included 447 (288 benign, 159 malignant) samples, all with a gold-standard histological diagnosis. Nuclei borders were manually highlighted from each slide, and morphological features (geometric and densitometric) were subsequently extracted for 41,324 nuclei. The model trained to classify these individually and employed radial basis function layers instead of more typical activation functions. A slide-level diagnosis was determined through a majority vote, which was based on either the number or percentage of nuclei classified as malignant with percentages more performant. The best model achieved 95.0% accuracy, 95.0% sensitivity and 95.1% specificity.

Elliott Range et al. (66) developed a system comprising two CNNs to predict malignancy from cytopathology WSIs, which eliminated the need to manually identify informative regions of cells as seen in other implementations. The study included 908 Papanicolaou-stained FNAs with a confirmed histopathological diagnosis. Nondiagnostic FNA and histopathology cases that were not either benign or malignant were excluded. The first CNN was used to locate informative regions of follicular groups; the second analysed these follicular groups and gave a TBS classification and associated diagnosis of benign or malignant. To develop the training set for the first CNN, a pathologist manually labelled informative regions. Noninformative regions were randomly selected areas of the scan; most of the scan is noninformative, so this had a high probability of providing regions that did not contain follicular cells. This CNN was trained, applied to each WSI, and used to extract the 1,000 identified regions identified with the highest probability of being informative; these 1,000 regions were then used to train the second CNN, which classified local regions as either benign or malignant and aggregated these into one global-level prediction for the final pathology. Both CNNs were trained using transfer learning and were based on VGG11. The system achieved an accuracy of 90.8%, sensitivity of 92.0%, specificity of 90.5%, and an AUROC of 0.932, the last of which was at the level of the original pathologist’s diagnosis (0.931). The authors found that combining their system with the original diagnosis improved the AUROC to 0.962, highlighting the potential of AI as an ancillary test.

Dov et al. (69) expanded the above work in Elliot Range et al. (66) by using weakly supervised learning for intervention-free thyroid-malignancy prediction from the same WSI dataset. Typically, cytopathology slides have a unique substructure with informative instances sparsely distributed throughout the slide, and the location and evaluation of these instances pose a challenge. They used a technique based on maximum likelihood estimation to propagate slide-level labels to local regions, using the propagated labels as ‘noisy’ labels, which led to an improved training strategy. They found that their two-stage algorithm – which was similar to that used in the previous work (66) – achieved expert-level human performance with an AUROC of 0.870 ± 0.017 for the best-performing implementation.

##### 2.1.2.2 Other Research Areas

Zhu et al. (67) [and Tao et al. (87)] worked towards efficient follicular cell segmentation from WSIs. The study included 43 WSIs (17 PTC, 26 benign), and 6,900 patches were cropped from 13 of these (all PTC) and used to train a DeepLabv3-based semantic segmentation model. The study did not employ transfer learning; the model was instead trained directly with the dataset. The authors added a classification branch that could designate patches as being an area of follicular cells, colloid or background; areas identified as containing follicular cells were then fed into the semantic segmentation structure, which improved efficiency – up to 93% of segmentation time was reduced by skipping the areas of colloid or background. When applied to 30 test WSIs, the hybrid segmentation model achieved a pixel accuracy of 99.3% in 49.5 seconds; it outperformed a fully convolutional network, U-Net and DeepLabv3, which achieved pixel accuracies of 96.3%, 96.3% and 97.7% in 370.8, 146.4 and 712.6 seconds, respectively.

Lin et al. (68) developed a fast-screening method for PTC segmentation from WSIs. The study included 131 Papanicolaou-stained FNA (120) and ThinPrep (11) PTC cytological slides, and ground truth annotations of PTC areas were provided by two expert pathologists. Each WSI was first preprocessed to discard areas of background, patched, and finally segmented by a CNN with a VGG16 backbone. Their system achieved 99% accuracy, 86% precision and 94% sensitivity, which outperformed U-Net and SegNet benchmarks, and could process WSIs 7.8x and 9.1x faster than those methods, respectively.

Dov et al. (71) further expanded on their earlier work by examining the clinical impact of an AI-based screening software, with their study measuring concordance between pathologist evaluation on 109 Papanicolaou-stained FNA biopsies (84 benign, 25 malignant) with and without using the tool. Labels were determined based on surgical pathology results. Initially, the pathologist evaluated the dataset independently, and after a washout period of 117 days, the same pathologist examined the same dataset with the assistance of the software. The system comprised a VGG11-based CNN screening algorithm trained as in the authors’ earlier work (66), with their software presenting a selection of 100 regions of interest to the pathologist through a graphical user interface, as well as a suggested prediction for malignancy. The concordance of results was measured with pairwise weighted kappa statistics; that for the assigned TBS category when the pathologist did and did not use the software was 0.924, indicating almost perfect concordance. When using the software, the average time spent per FNA was 81.6 seconds, and although similar statistics for the pathologist’s independent evaluation were not provided, this low case time highlights how effective a decision-support system can be at improving pathologist workflow.

The above presents compelling evidence that CNNs in particular can improve the thyroid cancer diagnosis pipeline. Most studies have focused on identifying PTC or predicting malignancy in general, but there is evidence to suggest that FTC and FA can be identified using nuclear features alone (60, 88, 89), a task that typically poses challenges during manual slide analysis, thus implementation of AI approaches have the potential to support cytology diagnoses that could be currently difficult or not possible Algorithms that obviate the requirement for manual patching and feature extraction are of particular interest (66): solutions that require less human intervention have greater potential to ease clinical workloads. The most recent paper by Dov et al. (71) is also notable, as it investigates the actual impact such AI methods can have once translated to the clinic, a welcome step forward from the more commonly observed retrospective analysis.

### 2.2 Histopathology

**Table 3** summarises research published since 2017 that applies AI to thyroid cancer histopathology. As with the research for cytopathology, the aims and datasets of each study vary, as do the levels at which the evaluation metrics are calculated, so the results are not directly comparable but have nevertheless been provided.

**Table 3:** A summary of recent research applying AI to histology specimens.

Study	Year	Aim	Technique	Level	Sample Size	Reported Metrics	Results
Jothi and Rajam (90)	2017	PTC vs normal thyroid	Ensemble learning with two support vector machines and a closest-matching-rule classifier	Image	219	Accuracy Sensitivity Specificity	99.5% 100% 98.6%
Wang et al. (91)	2019	Multiclassification of thyroid nodules	CNN	Slide	806	Accuracy	98.4%
				Image	11,715	Accuracy	97.3%
Tsou and Wu (92)	2019	Predict *BRAF* or *RAS* mutational status	CNN	Slide	103	Accuracy	95.2%
				Image	2,595	AUROC	0.951
Dolezal et al. (93)	2020	Classify NIFTP, PTC-EFG, and PTC as *BRAF*- or *RAS*-like	CNN	Slide	612	–	–
		Predict *BRAF-RAS* score and use it to discriminate NIFTP status		Slide	497	AUROC	0.99
Liu et al. (94)	2021	PTC vs normal thyroid	CNN	Image	2,772	Accuracy	98.6%
Esce et al. (95)	2021	Identify lymph nodal metastases	CNN	Slide	174	Sensitivity Specificity AUROC	94% 100% 0.964
El-Hossiny et al. (96)	2021	Multiclassification of thyroid nodules	Cascaded CNNs	Image	18,653	Accuracy	94.7%
Han et al. (97)	2021	PTC vs normal thyroid	CNN	Image	16,500	Sensitivity Specificity	95.8% 95.1%
Anand et al. (98)	2021	Predict *BRAF* mutational status	Weakly supervised CNN	Slide	529	AUROC	0.98
Böhland et al. (99)	2021	PTC-like (PTC, NIFTP and FV-PTC) vs non-PTC-like (FA, FTC)	CNNs and machine learning algorithms applied to two datasets	Slide	156	Accuracy	89.7%
				Slide	133	Accuracy	83.5%
Deng et al. (100)	2022	PTC vs non-PTC	Ensemble of a CNN and random forest	Image	610	Accuracy Sensitivity Specificity AUROC	93.8% 85.9% 97.2% 0.982
Stenman et al. (101)	2022	Quantification of tall cells in PTC	Two CNNs	Image	2,970	Sensitivity Specificity	93.7% 94.5%

PTC, papillary thyroid carcinoma; CNN, convolutional neural network; BRAF, RAS, gene types; AUROC, area under the receiver operating characteristic curve; NIFTP, noninvasive follicular thyroid neoplasm with papillary-like nuclear features; PTC-EFG, papillary thyroid carcinoma with extensive follicular growth; FV-PTC, follicular variant of papillary thyroid carcinoma.

Older papers that implement less advanced approaches include Wang et al. (89), Ozolek et al. (88), Kim et al. (102) and Jothi and Rajam (103).

#### 2.2.1 Machine learning-based methods

##### 2.2.1.1 Classification of carcinomas

Jothi and Rajam (90) implemented a system to differentiate PTC from normal thyroid tissue. Images were manually acquired by a pathologist from 12 tissue samples (4 normal thyroid, 8 PTC), with 219 images taken in total (64 normal thyroid, 155 PTC). Nuclei were segmented automatically using particle swarm optimisation-based Otsu’s multilevel thresholding, and morphological and texture features were extracted from each nucleus. The classification was performed on individual nuclei, and an ensemble learning model comprising a linear SVM, a quadratic SVM, and a closest-matching-rule algorithm achieved 99.5% accuracy, 100% sensitivity and 98.6% specificity on an image level.

Histopathology research has progressed from utilising traditional ML approaches, as evidenced by the lack of studies over the last five years.

#### 2.2.2 Deep learning-based methods

##### 2.2.2.1 Classification of carcinomas

Wang et al. (91) employed transfer learning to train two CNNs – Inception-ResNet-v2 and VGG19 – to classify thyroid nodules into multiple groups (normal thyroid tissue, adenoma, nodular goitre, PTC, FTC, MTC and ATC). The dataset comprised 806 H&E-stained histological images labelled by two senior pathologists: each gave an overall class for the WSI as well as a specific area of interest that influenced the classification. Cases of disagreement were discarded, meaning that the CNNs were not tested on these more difficult borderline cases. Each WSI was automatically segmented into 15 patches of nuclei: the Laplacian of Gaussian filter was used to highlight the nuclei in the WSI, one nucleus was selected at random to be the centre of a patch of size 448 x 448 pixels, and if the patch contained greater than 10% of the nuclei in the original image, it was extracted. The final dataset comprised 11,715 patches. The VGG19 model achieved an average patch-level accuracy of 97.3% and slide-level accuracy of 98.4%; it classified all malignant patches with an accuracy of above 97% and performed worst at identifying normal thyroid tissue, although it only mistook this for other benign classifications (goitre or adenoma). Although in this study the slides were ‘carefully selected’, the majority of the misclassifications involving adenoma and goitre were attributed to a lack of relevant features in the segmented patches, a consequence of the automatic method employed.

Liu et al. (94) trained an Inception Residual CNN as a feature extractor and combined it with an SVM to classify PTC from benign thyroid tissue. The study included 693 H&E tissue samples (261 benign, 432 PTC) each imaged at four magnifications (4x, 10x, 20x, 40x), giving 1,044 and 1,728 in each group, respectively, and images at the different magnifications were evaluated both separately and collectively. The authors implemented a colour transform to map each image into the same colour space, reducing the difference between tissue specimens from different staining intensities. Their algorithm performed best on the 40x magnified set, on which it achieved 98.6% accuracy.

El-Hossiny et al. (96) developed a system of two cascaded CNNs to classify WSIs: the first classified the thyroid tumours into PTC, FTC and FA, and the second subtyped those classified as FTC into four different subclasses. The study included 24 WSIs (9 PTC, 10 FTC, 5 FA), which were segmented into 18,653 512x512 pixel patches; 5% overlap was added to each side to increase this to 564x564 pixels, and the patches were subsequently scaled to give a final size of 282x282 pixels. Individual patches were manually labelled by pathologists and, following standard image augmentation, were used to train the two CNNs. Their algorithm achieved an overall patch-level accuracy of 94.7%.

Han et al. (97) focused on the autoclassification of patches from WSIs and used a multi-magnification method to classify PTC and normal thyroid lesions: images were taken at both 20x and 40x magnification to allow a CNN to mimic the diagnostic process of pathologists, where images are examined at a lower magnification with any suspicious areas examined more closely with a higher magnification. An experienced pathologist identified areas of PTC and normal thyroid in 55 tissue slides; the final dataset comprised 16,500 images (7,928 normal tissue, 8,572 PTC). The authors incorporated active learning by developing an algorithm that could identify unlabelled samples with high uncertainty and therefore a high potential to be informative. They then employed a VGG-f-based CNN to highlight which regions within the 20x-magnified images were most discriminative before extracting these patches at 40x magnification and feeding these samples into another CNN. If one of these 40x-magnified patches was identified as PTC, the eight surrounding patches were also tested to confirm the diagnosis. The top performing algorithm achieved 95.8% sensitivity and 95.1% specificity on an image level.

Böhland et al. (99) tested two approaches of designating samples as PTC-like (NIFTP, follicular variant of PTC, PTC) and non-PTC-like (FA, FTC). The first was feature-based and involved nuclei segmentation with DL, feature extraction, and classification with ML algorithms; the second involved direct classification where the images were fed into a CNN without the intermediary steps. They tested the methods on two datasets: the Tharun and Thompson dataset, which contained manually selected H&E-stained images from 156 thyroid tumours that were classified by two pathologists with consensus on every case; and the Nikiforov Box A, which contained 133 images that were submitted by six institutions as potential EFV-PTC, with the idea to define NIFTP out of these. The Nikiforov Box A, therefore, was considered by the authors to contain many borderline cases and identified as a more difficult dataset to classify. For each of 147 images in the Tharun and Thompson dataset, ten smaller images without overlap were extracted from neoplastic areas; for the other nine, fewer patches were extracted, as the neoplastic area was not large enough to facilitate ten. The feature-based classification method achieved an accuracy of 89.7% and 83.5% on the Tharun and Thompson dataset and Nikiforov Box A, respectively, and the DL-based method an accuracy of 89.1% and 77.4%, respectively – at the level of expert pathologists.

Deng et al. (100) used a multimodal approach to classify PTC from non-PTC. The study included 610 H&E-stained pathology samples (426 PTC, 184 non-PTC) from which two senior pathologists selected regions of interest and made diagnoses; samples were excluded in cases of disagreement. One patch was selected from each sample, and a ResNet50-based CNN was trained on these patches following standard image augmentation. A random forest was trained on the accompanying text-based features from laboratory tests for both thyroid function and ultrasound examination. The models’ predictions were then combined, which resulted in 93.8% accuracy, 85.9% sensitivity and 97.2% specificity; notably, the ensembled system achieved better results than either the CNN or random forest in isolation.

##### 2.2.2.2 Prediction of genetic mutational status

Tsou et al. (92) used transfer learning to train a CNN based on the Inception-v3 model to classify PTCs into having either *BRAF^V600E^* or *RAS* mutations. Tumours with the BRAF^V600E^ mutation characterise PTC and the tall cell variant of PTC, whereas those with the RAS mutation characterise follicular variant of PTC, so the hypothesis was that features of the histopathology images could predict these genetic mutations. From 103 H&E-stained slides taken from The Cancer Genome Atlas (TCGA), an expert pathologist manually selected 2595 patches, giving 25 patches per slide on average. A patch was labelled only if the model’s predicted probability was above 0.8, and a slide was classified only if at least 80% of the patches derived from the slide favoured one classification. With this exclusion rule, the model achieved 95.2% accuracy on the test set.

Dolezal et al. (93) theorised that the BRS (*BRAF-RAS*) score could help in identifying NIFTPs and aid in therapy de-escalation as mentioned above. Two pathologists digitally annotated an internal dataset of 115 H&E-stained images with regions encircling tumours; these were subsequently extracted and used to train an Xception-based CNN to predict tumour subtype out of NIFTP, PTC with extensive follicular growth, and PTC. The CNN was tested on a dataset from TCGA comprising 497 images, and between NIFTP and PTC with extensive follicular growth, tumours were 8.5x more likely to have an NIFTP prediction if they had a positive BRS (RAS-like) score. They further hypothesised that a predicted BRS score could aid in classification and trained a model on the 497 TCGA slides before testing on the internal cohort. NIFTPs were near-universally predicted to have RAS-like BRS, and as a discriminator of NIFTP status, the predicted BRS had an AUROC of 0.99 when all samples were included and 0.97 when restricted to NIFTPs and PTC with extensive follicular growth – the former had a mean predicted BRS of 0.35 and the latter -0.49.

Anand et al. (98) used a weakly supervised neural network to predict *BRAF* mutational status – which is associated with worse clinical features and outcomes – without regional annotations, as expert knowledge for labelling informative regions in such a task is unreliable; indeed, the authors first tried a supervised learning approach but found its performance limited by definitive labels for regions that were irrelevant or ambiguous. They employed attention-based multiple-instance learning, which can extract informative regions in large images, by using a VGG16-based CNN with an added attention module. The model was trained on a dataset comprising tumours from 85 patients; for each patient, 1–3 malignant microarray spots and one microarray spot of normal tissue were available, and each spot was augmented 50 times using flips and rotations. The model was tested on 444 samples sourced from TCGA where the authors sampled tumour-only regions using another neural network trained to localise the tumour region – this was as the training dataset had a greater proportion of tumour samples – and achieved an AUROC of 0.98. The authors also took the output of the attention module to generate a heatmap of informative regions: a *BRAF* probability was assigned to non-overlapping patches of the spot image and smoothed using a Gaussian spatial filter, giving a visualisation of high-attention regions and their probability for being *BRAF*-positive or -negative. They found high concordance with the informative regions and features typically associated with the *BRAF* mutation, such as papillary histology and oncocytic cells, with such visualisations aiding the move towards interpretable AI.

##### 2.2.2.3 Other research areas

Esce et al. (95) used a CNN to predict the presence of nodal metastases, which have prognostic importance but are often not sampled during initial surgery. A total of 174 primary tumour samples were included – 104 with regional metastases and 70 without. Study pathologists manually annotated regions to test two methods: one with the regions including only the tumour, and a second including the tumour and a regional transition zone. Smaller image patches were randomly selected from within the annotated zones and used for training and analysis. The second method – which included the transition zone – could predict nodal metastases with 96.3% accuracy, 93.6% sensitivity and 100% specificity and outperformed the case when the WSIs were fed directly into the algorithm, which was attributed to poor preservation of some areas of the tissue sample.

Stenman et al. (101) trained a CNN to quantify the proportion of tall cells in PTC; the tall-cell variant of PTC correlates with less favourable outcomes, but the clinical definition (at least 30% of epithelial cells 2–3 times as tall as they are wide) results in substantial interobserver variability. Their study included 190 PTC samples: 70 from one hospital and 30 from TCGA were used for system development, and 90 sourced from another hospital were used for external validation. The system comprised two algorithms working in sequence – the first segmented areas of tumour tissue, and the second identified regions of tall cells within the tumour – and were trained using 2970 manually annotated regions of interest. When evaluated on the external set, their algorithm could detect tall cells with 93.7% sensitivity and 94.5% specificity.

In this research, CNNs have shown great potential for automatic diagnosis when applied to histopathology samples. In their study, Wang et al. (91) achieved multiclassification to high accuracy, and although their algorithm was limited by the exclusion of borderline cases, it was more granular than comparable studies focusing on, for example, identifying only PTC or malignancy. Furthermore, the algorithm that could identify discriminative regions within images in the study of Han et al. (97) has the potential to expedite pathologist workflow, although one limitation is that it used patches for input instead of working directly on WSIs. Notably, the four of the above studies sourced tissue samples from TCGA, compelling evidence for how open-source, multimodal datasets can facilitate new avenues for research. The work of Dolezal et al. (93) introduced a potential avenue for NIFTP identification, although it remains to be seen if such a technique could work on preoperative samples. Importantly, the introduction of the attention mechanism seen in the work of Anand et al. (98) is a further step towards explainability, the lack of which is a barrier to clinical integration.

## 3 Discussion

This review has identified a plethora of compelling evidence suggesting that AI can improve the cyto- and histopathology processing pipelines for thyroid cancer diagnosis and risk stratification. Current issues with thyroid FNA biopsies – including high interobserver variability (21, 22), a significant proportion returning indeterminate samples (11, 16), and the fact that some pathologies cannot be reliably classified using cytological criteria (76) – necessitate such improvements; histopathology analysis addresses these to an extent but requires surgical resection so cannot guide preoperative decisions. It is important to note that there is a substantial observer variation not only in thyroid cytology but also in thyroid histopathology (104–107). Additionally, given the chronic shortage of trained pathologists (25), technologies that can reduce the demand for clinicians’ time should be welcomed and readily adopted. The issue is pertinent given the unprecedented pressure under which health services must currently operate; exacerbated by the COVID-19 pandemic, the number of NHS patients awaiting elective care is at a record high of six million as of November 2021 (108). AI technologies must not, of course, be rushed through without proper evaluation; indeed, just how AI-based medical devices are evaluated has rightly come into question recently (109). Great care must be taken to ensure solutions are effective, transferable, robust and free of biases (110); the latter has, for example, caused notable issues with algorithms designed to detect skin cancer (111).

While AI-based methods certainly show promise within thyroid cancer, there is a lack of research into the clinical realisability of such methods, except for the study of Dov et al. (71). Future studies could aim to elucidate further the extent to which pathologists could rely on these systems for ancillary decision-making and guided investigation. Examining performance on a test set gives an idea of accuracy and transferability, but these models could certainly not be integrated to replace human-based pathological analysis immediately; rather, confidence must be developed gradually through the deployment of trust-building AI-enhanced workflows. Somewhat to that end, and certainly in line with the necessary recent emphasis on developing explainable AI, some of the more recent studies have investigated the areas that guide CNNs towards their classification (69, 97, 98); the production of informative-instance heatmaps could direct clinicians to discriminative areas and expedite their investigation. Thyroid pathology studies could take inspiration from recent advances in other areas of pathology (112, 113) to further develop these ideas. Multi-task architectures could also help in this respect: implementations could provide both a global classification and these heatmaps, and one could imagine an additional arm that utilises natural language processing, where AI is used to interact with human language, to provide a textual justification, all of which could foster clinical confidence in the diagnostic result. Further research is certainly welcome in this area: interpretability is important for establishing the necessary trust with clinicians and regulators, and exploration into the practicalities of how these technologies could improve routine examination would assist clinical adoption (40).

Notably, much of the past research has focused on binary classification and PTC given the latter’s predominance over other malignancies, but the examination of thyroid FNA and surgical specimens is significantly more nuanced than many past studies have addressed. The research conducted by Wang et al. (91) is one exception with the authors achieving high accuracy when attempting multiclassification, although a limitation was the exclusion of cases on which the study pathologists disagreed. Additionally, given that laboratory datasets are often heterogeneous – different labs often use different methods of cell and tissue preparation, fixation, staining and imaging – algorithms must typically be adapted to suit the clinical needs of each lab (99). To be truly generalisable and robust, a method should be capable of handling these institutional differences. Some studies have begun to address this – by, for example, examining datasets with multiple stains and from different institutions (60, 62, 99), or by implementing a colour transform for different staining intensities (94) – but future studies that include diverse multicentre datasets and demonstrate high performance in a process- and equipment-agnostic manner are encouraged. Federated learning and domain adaptation are two avenues that warrant further investigation in this respect: they have been applied in other areas of pathology to improve interlaboratory transferability (114, 115).

Additionally, although much of the past research has focused on the diagnostic classification of cyto- and histopathology specimens, other avenues that investigate different areas of thyroid cancer therapy certainly exist. The prediction of nodal metastases in the work of Esce et al. (95) is an example with real clinical relevance: the authors themselves posit the idea of a real-time algorithm that could guide a decision on whether or not to perform a central neck dissection. Furthermore, although the classification of NIFTPs has been studied to an extent (64, 93, 99), it has proven a challenging area of research and, given the significant potential for reliable NIFTP identification to aid in therapy de-escalation, this necessitates further studies into how AI technologies could improve current processes.

Many of the methods reviewed here require representative labels to be assigned to the extracted patches. The models themselves often do not take an entire WSI as input; instead, they make predictions on these smaller segmented images before aggregating these individual predictions into a patient- or slide-level prediction. To generate such a training set of labelled patches – where WSIs are generally examined manually for informative regions by pathologists – is laborious and costly work that requires expert knowledge. In some studies, researchers have automatically patched areas of the WSIs and have circumvented the requirement for manual annotation of the segmented images by assigning the original WSI diagnosis (91) – a consequence of such an approach is that representative features of the slide-level diagnosis will not exist in all patches, as it is common for cyto- or histopathology WSIs to contain areas of both normal tissue and the pathology should there be one. Furthermore, to implement some of the current models in clinical practice, a pathologist may have to manually identify the representative regions to use as inputs. As explored above, some studies have addressed this with new approaches, including automatic informative region identification, active learning and weakly supervised multiple-instance learning (66, 67, 69, 87, 97, 98); future studies could further research such techniques that bring diagnostic pipelines closer to true automation.

In summary, while AI has shown great potential to improve the thyroid cancer diagnosis pipeline, current research suffers from several limitations: a lack of focus on clinical integration of AI-based methods and how they can improve workflows in practice; utilisation of patch-level labels, for which training set acquisition can be laborious and costly; and a focus on binary classification and PTC, as opposed to multiclassification of all subtypes. These limitations highlight avenues for future research: evaluate the practical potential of an algorithm to assist clinical decision-making; expand the current research on explainability, which can help to build trust with clinicians and regulators; further investigate techniques that require only a slide-level label and can direct pathologists to regions of interest; and examine large multicentre datasets to develop robust techniques that are agnostic to the processes and equipment of individual laboratories.

## Author contributions

GS: contributed to the writing and revision of the manuscript, read and approved the manuscript. LB: contributed to the revision of the manuscript, read and approved the manuscript. HR and PD: read and approved the manuscript. EM: conceived, designed and supervised the project, contributed to the writing, editing and revision of the manuscript, read and approved the manuscript. All authors contributed to the article and approved the submitted version.
